# Supplementary material for: Identification of collaborative cross mouse strains susceptible to chlamydial induction of hydrosalpinx
Source: Infect Immun. 2026 Feb 17;94(3):e00744-25. doi: 10.1128/iai.00744-25 (PMC12974144; doi:10.1128/iai.00744-25)

Supplementary figure

Fig. 1S. Gross pathology images of the female genital tracts from five strains of CC mice on day 56 after intravaginal inoculation with SPG buffer alone.

The five CC strains of mice, as indicated on the left of the corresponding images (n=3 to 5), were intravaginally inoculated with SPG alone as described in the legend of Fig. 1. On day 56 after inoculation, all mice were sacrificed to observe genital tract pathology, including uterine horn dilation (white arrowheads) and oviduct hydrosalpinx (white arrows). The image from each mouse is presented with the whole genital tract on the left and the magnified oviduct/ovary portion on the right. The individual mouse ID is displayed in the lower-left corner of the corresponding image. Note that no significant hydrosalpinx was detected in any of the control CC mice, while uterine horn dilation was detected.

Fig.1S, Wu et al

Gross Pathology Images of SPG-inoculated CC mice

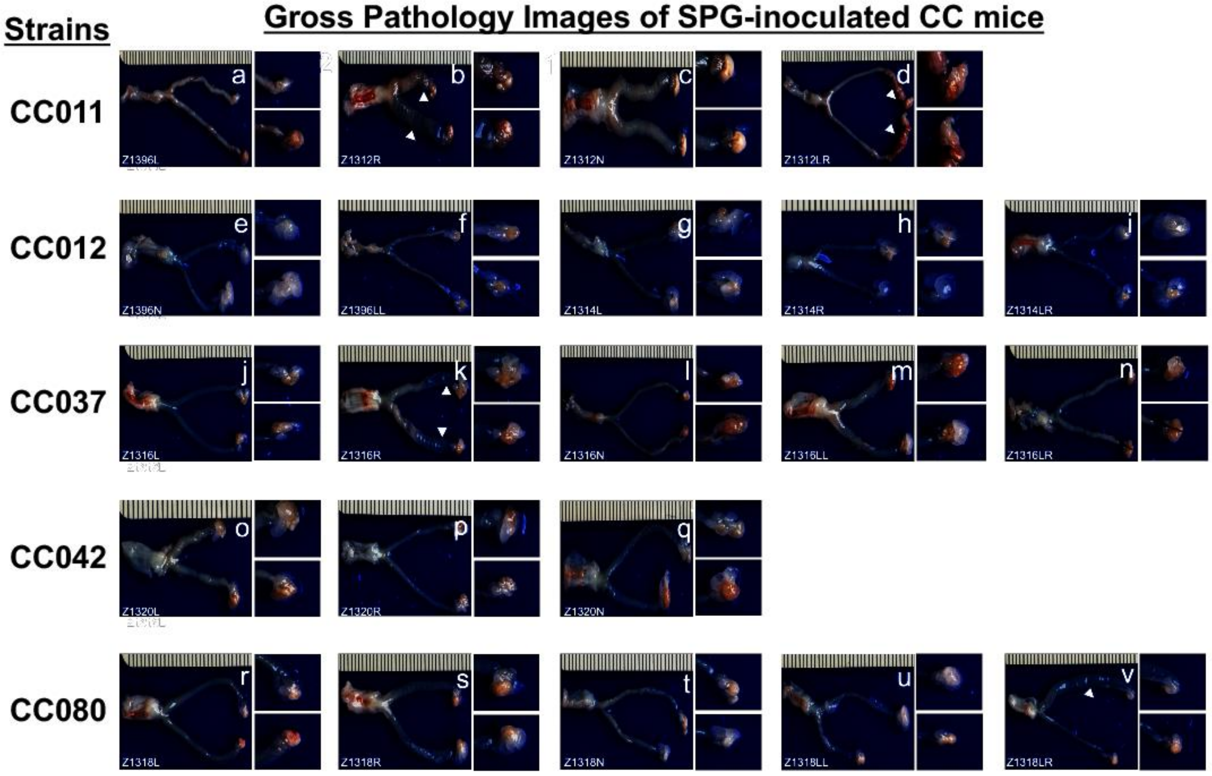

Supplement: Fig. S1 — Gross pathology images of the female genital tracts from five strains of CC mice on day 56 after intravaginal inoculation with SPG buffer alone. [file iai.00744-25-s0001.pdf]
